# An AI-derived inflammation-metabolism ecosystem signature reveals ATP2A2 as a microenvironment-remodeling driver of immune evasion in lung adenocarcinoma

**DOI:** 10.3389/fcell.2026.1971779

**Published:** 2026-10-14

**Authors:** Xiujuan Hu, Guoxin Zhang, Yue Zhang, Yong Zhao, Fang Zhou, Guanhua Li

**Affiliations:** 1 Department of Respiratory and Critical Care Medicine, Tianjin Chest Hospital, Tianjin University, Tianjin, China; 2 Department of Pathology, Tianjin Medical University Cancer Institute and Hospital, National Clinical Research Center for Cancer, Tianjin’s Clinical Research Center for Cancer, Tianjin, China; 3 Department of Thoracic Surgery, Tianjin Chest Hospital, Tianjin University, Tianjin, China

**Keywords:** ATP2A2, immunotherapy resistance, inflammation-metabolism signature, lung adenocarcinoma, tumor immune microenvironment

## Abstract

**Background:**

Chronic inflammation and metabolic dysregulation are increasingly recognized as interconnected drivers of tumorigenesis and immune evasion, yet their combined prognostic and mechanistic relevance in lung adenocarcinoma (LUAD) remains poorly defined.

**Methods:**

A curated 117-gene set representing systemic inflammation-metabolism dysregulation was obtained from MSigDB. Core prognostic genes were identified through differential expression analysis and univariate Cox regression. Using TCGA-LUAD as the training cohort and six independent GEO cohorts for external validation, 101 machine learning algorithm combinations were systematically evaluated to construct a robust prognostic model. Model performance was assessed by C-index, time-dependent ROC, and PCA. Immune characteristics were analyzed using TCIA, ESTIMATE, and TIMER-based deconvolution. ATP2A2, identified as the gene most strongly correlated with risk score, underwent multi-cohort transcriptomic and immunohistochemical validation, *in vitro* functional characterization, and mechanistic investigation via IOBR-integrated immune profiling. Single-cell RNA sequencing and multiplex immunofluorescence provided cellular-resolution verification of microenvironmental associations.

**Results:**

The RSF + SuperPC model achieved optimal performance and effectively stratified patients into high- and low-risk groups with significantly divergent survival across all seven cohorts. High-risk tumors showed reduced immune/stromal infiltration and elevated tumor purity. ATP2A2 was consistently overexpressed in tumor tissue, correlated with poor differentiation, advanced stage, and shorter survival, and predicted immunotherapy resistance. Mechanistically, high ATP2A2 expression was associated with suppressed immune cell infiltration, attenuated chemokine signaling, impaired cancer-immunity cycle activity, and an “immune-desert” phenotype confirmed by single-cell and spatial analyses.

**Conclusion:**

This study establishes a validated inflammation-metabolism-based prognostic signature in LUAD and identifies ATP2A2 as a key driver linking tumor aggressiveness to immunosuppressive microenvironment remodeling, positioning it as a promising prognostic biomarker and potential therapeutic target for overcoming immunotherapy resistance.

## Introduction

Lung cancer remains the leading cause of cancer-related mortality worldwide. In the United States alone, more than two million new cancer cases were projected in 2024, with lung cancer continuing to top the list of cancer deaths (Siegel et al., 2024), and globally, lung cancer ranks among the highest in both incidence and mortality across all malignancies (Bray et al., 2024). Lung adenocarcinoma (LUAD), the predominant histological subtype of non-small cell lung cancer, continues to pose formidable clinical challenges—including treatment resistance, recurrence, and distant metastasis—despite the introduction of targeted therapies and immune checkpoint blockade, which have yielded meaningful benefit in only a subset of patients. Part of this therapeutic impasse stems from the recognition that tumors are not isolated cellular entities but complex systems in continuous, co-evolving interaction with their microenvironment and the host’s systemic physiological state.

Chronic inflammation and metabolic dysregulation, two classical drivers of malignancy, have recently been reconceptualized as a pair of deeply intertwined, mutually reinforcing hallmarks of cancer biology (Hanahan, 2022). Persistent inflammatory signaling can recruit immunosuppressive cell subsets, remodel the extracellular matrix, and sustain a pro-tumorigenic cytokine network that fosters immune evasion. Concurrently, metabolic reprogramming in both tumor cells and their surrounding immune cells not only fulfills the bioenergetic and biosynthetic demands of uncontrolled proliferation but also directly suppresses effector lymphocyte function through nutrient competition and metabolite-mediated signaling, thereby entrenching an immunosuppressive phenotype at the local microenvironmental level (Zhang et al., 2024).

Among the molecular machinery operating at the intersection of inflammation and metabolism, intracellular calcium signaling networks have drawn increasing attention. The spatiotemporal dynamics of calcium signaling serve as a core regulator of cellular metabolic activity and are deeply implicated in inflammasome activation and the execution of immune effector functions; their dysregulation has been shown to promote tumor cell survival, migration, and therapeutic resistance through multiple downstream pathways (Wu et al., 2021). As a key molecule within this network, aberrant expression of the sarco/endoplasmic reticulum calcium ATPase SERCA2 family has been independently reported across several tumor types: silencing of ATP2A2 has been identified as an early and frequent event during oral carcinogenesis, whereas in diffuse astrocytomas, ATP2A2 expression correlates significantly with tumor grade and patient survival—suggesting that this gene may play complex, and even divergent, roles depending on tumor context, with its specific function in lung adenocarcinoma remaining to be defined.

Translating the concept of systemic inflammation-metabolism dysregulation into a molecular tool of genuine clinical utility still faces several core challenges. First, existing studies have largely focused on isolated genes within either inflammatory or metabolic pathways in isolation, lacking a systematic characterization of the gene sets at their intersection. Second, although numerous prognostic gene signatures have been reported in LUAD, the generalizability of most models across platforms remains constrained by reliance on a single modeling algorithm and limited validation cohorts; systematically pairing and cross-validating over a hundred machine learning algorithms across multiple independent cohorts has proven to be an effective strategy for improving model robustness and reproducibility (Liu et al., 2022). Third, and most critically, identifying biologically interpretable hub genes from within such integrative signatures—and elucidating how they drive malignant phenotypes and immune microenvironment remodeling—represents a translational step from “association” to “mechanism” that remains largely unexplored in the current literature.

Meanwhile, although immune checkpoint blockade has reshaped the therapeutic landscape of LUAD—exemplified by atezolizumab’s significant survival benefit in previously treated non-small cell lung cancer patients (Rittmeyer et al., 2017)—a substantial proportion of patients exhibit primary or acquired resistance. The generation of an effective antitumor immune response depends on a sequential cascade of steps collectively termed the “cancer-immunity cycle,” encompassing antigen release, presentation, T cell priming, trafficking, and killing; disruption at any step can result in treatment failure (Chen and Mellman, 2013). One core mechanism underlying this resistance is the dynamic transition of the tumor microenvironment from an immune cell-rich “hot” phenotype to an immune-cold, immune-desert phenotype characterized by sparse infiltration and activated immunosuppressive pathways (Wu et al., 2024). Tools such as the ESTIMATE algorithm, which infers tumor purity and stromal/immune cell fractions from expression profiles (Yoshihara et al., 2013), the TIMER database integrating multiple deconvolution methods for immune infiltration estimation, and single-cell transcriptomic profiling for dissecting tumor and microenvironmental heterogeneity at cellular resolution (Kim et al., 2020), now enable systematic evaluation of how specific gene expression correlates with immune microenvironment remodeling across multiple levels of resolution—providing powerful tools for dissecting the molecular mechanisms by which inflammation-metabolism dysregulation drives immune evasion.

Building on these gaps in current understanding, this study aims to systematically characterize the molecular features and clinical significance of a systemic inflammation-metabolism dysregulation-related gene set in LUAD, to identify core genes of both prognostic value and mechanistic relevance through integrated multi-omics analysis and machine learning strategies, and to further investigate—across cellular, tissue, and single-cell levels of resolution—the potential mechanisms by which these genes drive tumor immune microenvironment remodeling and immunotherapy resistance, thereby providing a novel theoretical basis for precision molecular subtyping and optimized immunotherapeutic strategies in LUAD.

## Methods

### Study design and data sources

This study integrated public bulk transcriptomic, genomic, clinical, immunotherapy and single-cell datasets with immunohistochemical and *in vitro* validation to develop an inflammation-metabolism-related prognostic model for lung adenocarcinoma (LUAD) and to investigate ATP2A2 as a candidate mediator of tumour progression and immune remodelling. The TCGA-LUAD cohort was used for model development. Six independent Gene Expression Omnibus (GEO) cohorts (GSE13213 (Tomida et al., 2009), GSE26939 (Wilkerson et al., 2012), GSE29016 (Staaf et al., 2012), GSE30219 (Rousseaux et al., 2013), GSE31210 (Yamauchi et al., 2012) and GSE42127 (Hight et al., 2020)) were used for external prognostic assessment. Additional GEO cohorts, the OAK and POPLAR immunotherapy cohorts, and the single-cell RNA-sequencing dataset HRA001130 were used for gene-level and mechanistic analyses. Samples lacking the expression or clinical variables required for a given analysis were excluded from that analysis.

Gene-expression values were transformed as specified for each dataset. TCGA and GTEx expression values used for tumour-normal comparisons were analysed as log2 (TPM +1). GEO probe identifiers were mapped to gene symbols using the corresponding platform annotations; when multiple probes mapped to the same gene, their expression values were averaged. Within-cohort Z-score or min-max transformation was used only for the cross-platform visualizations and clinical subgroup analyses indicated below. Survival time was converted to years before plotting and time-to-event modelling.

### Inflammation-metabolism gene set and genomic characterization

The Molecular Signatures Database (MSigDB) gene set ‘Systemic inflammation-metabolism dysregulation and cancer’, comprising 117 genes, was obtained through the GSEA portal. Somatic mutation data from 289 TCGA-LUAD tumours were summarized with the maftools R package (Mayakonda et al., 2018) to determine mutation frequencies, variant classifications and mutation types. Genomic positions were defined using the GRCh38/hg38 assembly. Copy-number data were obtained from the UCSC Xena platform and used to display amplification and deletion patterns at the chromosomal locations of the selected genes.

### Identification of core prognostic genes

Expression of the 117 candidate genes was compared between TCGA-LUAD tumours and GTEx normal lung samples using two-sided Wilcoxon rank-sum tests, with Benjamini–Hochberg adjustment for multiple testing. Genes with an adjusted P value below 0.05 were considered differentially expressed. In parallel, univariable Cox proportional-hazards models were fitted in TCGA-LUAD to test associations with overall survival. Genes associated with survival at P < 0.05 and differentially expressed between tumour and normal lung were retained. This intersection yielded 16 core genes: ADM, PFKP, MT2A, PLAUR, CDA, PSMA3, TNFRSF12A, UBE2L6, LGALS3, ATOX1, ATP2A2, HSPA5, CP, IL13RA1, CFB and F10.

### Machine-learning model development and validation

TCGA-LUAD samples with complete expression and survival data (n = 516) constituted the training cohort. The six external GEO cohorts comprised 714 samples, giving 1,230 samples across the seven prognostic cohorts. Batch effects in the 16-gene expression matrix were adjusted using ComBat in the sva R package (Leek et al., 2012), and principal component analysis (PCA) was used to examine cohort structure before and after correction. A total of 101 survival-learning algorithms and algorithm combinations were evaluated. Candidate methods included least absolute shrinkage and selection operator, ridge regression, elastic net, stepwise Cox regression, CoxBoost, random survival forest (RSF), supervised principal components (SuperPC), survival support-vector machines, gradient boosting machines and partial least-squares Cox regression. Models were fitted in TCGA-LUAD and evaluated by the concordance index (C-index) in each external cohort. The mean C-index across the six validation cohorts was used to rank models; the RSF + SuperPC model had the highest mean validation C-index and was selected for downstream analysis.

The selected model generated a continuous risk score for each patient. Maximally selected rank statistics, implemented in survminer, were used to define high- and low-risk groups. PCA of the model genes was used to visualize separation between risk groups.

### Risk score, tumour immunogenicity and the immune microenvironment

Immunophenoscores (IPS) for TCGA-LUAD were obtained from The Cancer Immunome Atlas. IPS reflects the immunogenicity level of tumors, with higher scores indicating greater potential for immunotherapy efficacy. The four IPS categories defined by CTLA4 and PD1 status were compared between risk groups using two-sided Wilcoxon rank-sum tests. These categories were treated as *in silico* estimates of relative immunogenicity under the corresponding checkpoint-inhibition settings rather than as observed treatment responses.

The ESTIMATE algorithm was applied to TCGA-LUAD expression data to calculate StromalScore, ImmuneScore and ESTIMATEScore and to infer tumour purity. Associations between continuous risk score and these metrics were tested using Spearman correlation. Immune-cell abundance estimates generated by TIMER, CIBERSORT, CIBERSORT-ABS, QUANTISEQ, MCPcounter, xCell and EPIC were obtained through TIMER2.0 and compared between high- and low-risk groups.

### Prioritization and multi-cohort validation of ATP2A2

Within TCGA-LUAD, expression of the model genes was correlated with the continuous RSF + SuperPC risk score using two-sided Pearson correlation. Scatter plots showed ordinary least-squares fits with 95% CIs. ATP2A2 was prioritized for further investigation on the basis of its association with the model risk score and its clinical and biological relevance.

ATP2A2 expression was compared between tumour and normal or adjacent lung tissues in TCGA-LUAD and GSE68465, GSE11969, GSE19188, GSE31210 and GSE63459 using two-sided Wilcoxon rank-sum tests. Within each GEO cohort, log2-transformed expression was Z-score standardized for cross-cohort visualization. Associations between ATP2A2 expression and primary tumor category, histological differentiation, and pathological stage were evaluated across GSE30219, TCGA-LUAD, GSE68465, and GSE31210 cohorts.

### Immunohistochemistry and quantitative image analysis

Formalin-fixed, paraffin-embedded LUAD and matched adjacent or normal lung tissues were cut into 4-μm sections and baked at 60 °C for 1 h. Sections were deparaffinized in xylene, rehydrated through graded ethanol and subjected to heat-induced antigen retrieval in citrate buffer (pH 6.0) or EDTA buffer (pH 9.0). Endogenous peroxidase was blocked with 3% hydrogen peroxide for 10 min, and nonspecific binding was blocked with bovine serum albumin for 30 min at room temperature. Sections were incubated with an ATP2A2 primary antibody overnight at 4 °C, followed by an HRP-conjugated secondary antibody for 30 min at room temperature. Staining was developed with 3,3′-diaminobenzidine, counterstained with haematoxylin, dehydrated, cleared and mounted. Histological diagnosis and stage were confirmed by pathologists, and staining was assessed without knowledge of the clinical group.

### ATP2A2 overexpression and transwell assays

A549 cells were transfected with ATP2A2 overexpression plasmid or empty vector using lipid-based reagent, stable clones were selected with antibiotic, and ATP2A2 overexpression was verified by immunoblotting or qPCR. Cells in logarithmic growth were converted to single-cell suspensions and seeded in the upper chamber of Transwell inserts. Uncoated membranes were used for migration assays and basement-membrane-matrix-coated membranes for invasion assays; medium containing 10% serum in the lower chamber served as the chemoattractant. After 24–48 h, cells remaining on the upper membrane were removed, and migrated or invaded cells were fixed in 4% paraformaldehyde and stained with crystal violet.

Five randomly selected fields per membrane were imaged under identical settings and counted using ImageJ. Field counts were averaged to obtain one value for each biological replicate. Three independent biological replicates were analysed per group. Migration and invasion were plotted separately as bar graphs showing the mean ± s.d. Together with individual replicate values, and ATP2A2-overexpressing and control cells were compared using a two-sided unpaired t-test.

### Immunotherapy cohorts and ATP2A2-associated immune features

ATP2A2 expression and progression-free survival (PFS) and overall survival data were analysed in patients receiving immune-checkpoint inhibitors in the OAK (n = 257) and POPLAR (n = 59) cohorts. Within each cohort, patients were stratified at the optimal cutoff value of ATP2A2 expression into high- and low-expression groups. Patients were stratified at the optimal cutoff value of ATP2A2 expression into high- and low-expression groups.

ESTIMATE scores and inferred tumour purity were calculated in OAK and TCGA-LUAD, and their associations with ATP2A2 expression were tested using two-sided Pearson correlation. In TCGA-LUAD, immune infiltration was assessed using the seven TIMER2.0 algorithms listed above. IOBR was used in OAK and TCGA-LUAD to integrate ESTIMATE, MCPcounter, EPIC and QUANTISEQ scores. For each immune feature, correlations with continuous ATP2A2 expression were estimated using Pearson correlation, and high- versus low-expression groups were compared using two-sided Wilcoxon rank-sum tests. Z-score-normalized scores were used for heatmap display only.

Chemokines, chemokine receptors, immunoinhibitory molecules, immunostimulatory molecules and MHC-associated genes were obtained from the immunomodulatory gene sets implemented in IOBR. Pearson correlations between ATP2A2 and each immunomodulatory gene were calculated within each cohort. Activity of the seven steps of the cancer-immunity cycle was quantified by single-sample gene-set enrichment analysis and correlated with ATP2A2 expression.

### Single-cell RNA-sequencing analysis

The HRA001130 single-cell RNA-sequencing dataset was processed in Seurat (Butler et al., 2018). Low-quality cells were removed on the basis of detected-gene counts, unique molecular identifier counts and mitochondrial transcript percentage. After normalization, highly variable features were identified and scaled. PCA was performed on the variable-gene matrix, a shared-nearest-neighbour graph was constructed, and cells were clustered using FindClusters and visualized with UMAP. Cell identities were assigned from established lineage markers, including AGER and SFTPC for alveolar epithelial cells, CD3D and CD3E for T cells, C1QA and C1QB for macrophages, PECAM1 and VWF for endothelial cells, and COL1A1 for fibroblasts.

Samples were stratified into ATP2A2-high and ATP2A2-low groups using sample-level ATP2A2 expression. Cell numbers and proportions were summarized by cell type and sample, and compositional differences were evaluated at the sample level. Marker expression and the fraction of cells expressing each marker were displayed in dot plots. UMAPs and stacked composition plots were used for visualization; individual cells were not treated as independent biological replicates in between-group statistical tests.

### Multiplex immunofluorescence

Multiplex immunofluorescence was performed on human LUAD sections using DAPI to label nuclei, ATP2A2, pan-cytokeratin to identify epithelial or tumour cells, and CD8 to identify cytotoxic T cells. Multispectral images were used to examine the spatial distribution of pan-cytokeratin-positive tumour regions and CD8-positive cells in areas with low or high ATP2A2 staining. Images from the different channels were acquired and processed using the same settings within each comparison.

### Statistical analysis

All tests were two-sided unless stated otherwise. Continuous variables were compared using the Wilcoxon rank-sum test for two independent groups, an appropriate paired test for matched specimens, or the Kruskal–Wallis test for more than two groups. Associations between continuous variables were evaluated using Pearson or Spearman correlation as specified above. For survival analysis, Kaplan-Meier curves and two-sided log-rank tests were used for group comparisons, time-dependent ROC curves were calculated at 1-, 3-, and 5-year time points, and all survival time was expressed in years. Effect sizes were reported as HRs with 95% CIs from Cox proportional-hazards models. For analyses involving multiple genes, immune features or pairwise contrasts, P values were adjusted using the Benjamini–Hochberg method. P < 0.05 or adjusted P < 0.05, as appropriate, was considered statistically significant. Analyses were performed in R using the packages described above; plots were generated with ggplot2, ggpubr and survminer.

## Result

### Inflammation-metabolism screening identifies survival-associated genes

We first characterized a curated set of 117 genes linked to systemic inflammation-metabolism dysregulation. In TCGA-LUAD, 206 of 289 tumours (71.28%) carried at least one somatic alteration in this gene set. PLG, MET and HNF4A were the most frequently mutated genes, with mutation frequencies of 10%, 7% and 6%, respectively (Figure 1a; Supplementary Figure S1). We next combined tumour-normal differential-expression analysis with univariable Cox regression to focus the screen on genes that were both dysregulated in LUAD and associated with overall survival. This analysis retained 16 genes: ADM, PFKP, MT2A, PLAUR, CDA, PSMA3, TNFRSF12A, UBE2L6, LGALS3, ATOX1, ATP2A2, HSPA5, CP, IL13RA1, CFB and F10. F10, CFB, MT2A and ADM were expressed at lower levels in tumours than in normal lung, whereas the other 12 genes were upregulated (Figure 1b). Fifteen genes had hazard ratios above one in the univariable analyses; F10 was the only gene associated with lower risk (HR = 0.86; Figure 1c). The 16 genes were distributed across multiple chromosomes and showed both copy-number gains and losses. F10 had the highest deletion frequency, whereas gains were prominent for several genes, including TNFRSF12A, LGALS3 and PSMA3 (Figure 1d). These analyses defined a compact inflammation-metabolism panel that linked transcriptional dysregulation with survival in LUAD.

**FIGURE 1:**
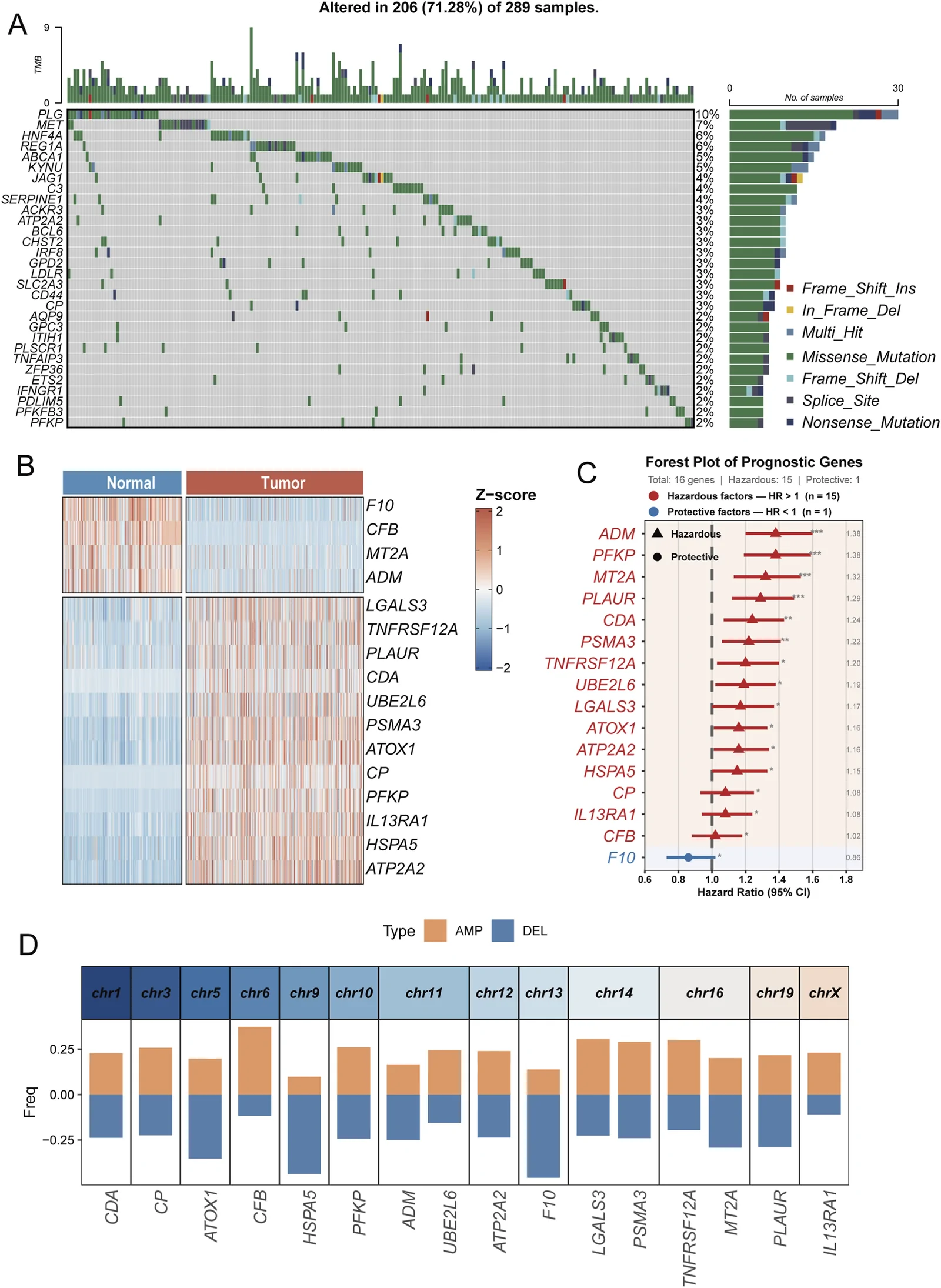
Mutational landscape, differential expression, prognostic value, and copy number variation of inflammation-metabolism-related genes in LUAD. **(A)** Waterfall plot showing the mutational landscape of the 117 inflammation-metabolism-related genes across 289 TCGA-LUAD tumor samples. Each column represents an individual sample; each row represents a gene. Genes are ranked by mutation frequency, with PLG, MET, and HNF4A among the most frequently mutated. Color-coded bars indicate different variant classification types. Top and side bar plots denote per-sample and per-gene mutation burden, respectively. **(B)** Heatmap/box plot comparing the expression levels of the 16 core genes between TCGA-LUAD tumor samples and GTEx normal lung tissue samples. F10, CFB, MT2A, and ADM were downregulated in tumor tissue, whereas the remaining 12 genes were significantly upregulated in tumor tissue relative to normal lung. **(C)** Forest plot illustrating the hazard ratios (HR) with 95% confidence intervals and P values derived from univariate Cox regression analysis for the 16 core genes in the TCGA-LUAD cohort. Fifteen genes were identified as risk factors (HR > 1), while F10 was the sole protective factor (HR = 0.86). **(D)** Chromosomal map depicting the genomic locations of the 16 core genes and their copy number variation status in TCGA-LUAD. F10 (chromosome 13) was subject to copy number loss, whereas TNFRSF12A, LGALS3, and PSMA3 (chromosomes 14 and 16) displayed copy number gain.

### RSF + SuperPC model shows robust prognostic performance

We used TCGA-LUAD for model development and six independent GEO cohorts for external assessment. Among 101 survival-learning algorithms and algorithm combinations, RSF + SuperPC achieved the highest mean C-index across the six validation cohorts (mean C-index = 0.648; Figure 2a). Following batch correction, PCA showed substantial overlap among the seven cohorts, with no dominant separation by data source (Figure 2b; Supplementary Figure 2a). The RSF + SuperPC score separated patients into groups with different overall survival in the TCGA training cohort (HR = 2.10, 95% CI 1.58–2.81; log-rank P < 0.0001) and in each GEO cohort (Figures 2c–i). The external-cohort HRs ranged from 1.93 in GSE13213 to 26.82 in GSE31210. Estimates were less precise in the smallest cohort, GSE29016 (HR = 6.51, 95% CI 0.88–48.16), despite a log-rank P value of 0.035. Thus, the direction of the survival difference replicated across datasets, although its magnitude and precision varied between cohorts. Time-dependent discrimination was similarly heterogeneous. In TCGA-LUAD, the 1-, 3- and 5-year AUCs were 0.618, 0.643 and 0.638, respectively (Supplementary Figure 2b). Across the GEO cohorts, reported AUCs ranged from 0.527 to 0.814 (Figures 3A–F). Performance was strongest for 1-year survival in GSE13213 (AUC = 0.814), for 1-, 3- and 5-year survival in GSE30219 (0.797, 0.713 and 0.738), and for 3- and 5-year survival in GSE31210 (0.768 and 0.789). By contrast, several time points in GSE13213, GSE29016 and GSE42127 showed only modest discrimination. PCA based on the 16 genes partly separated the risk groups in all six validation cohorts, but the degree of overlap differed across datasets (Figures 3g–l; Supplementary Figure 2c). Collectively, the model consistently stratified survival risk, whereas absolute predictive accuracy depended on cohort and time horizon.

**FIGURE 2:**
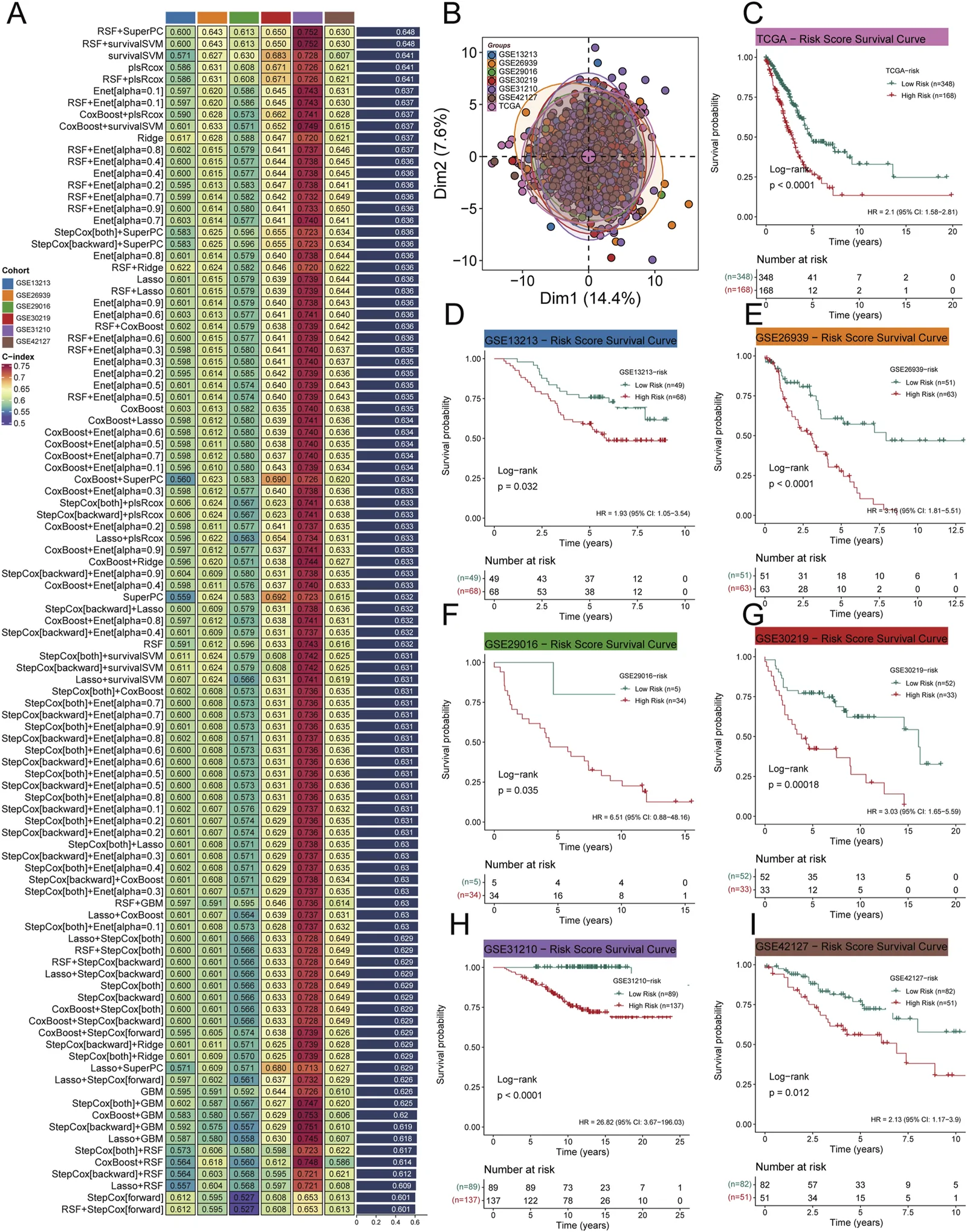
A machine learning-based prognostic risk model constructed from 16 core genes and its validation across multiple cohorts. **(A)** Heatmap of C-index values for 101 machine learning algorithms and their combinations, evaluated in the TCGA training cohort and six GEO validation cohorts (GSE13213, GSE26939, GSE29016, GSE30219, GSE31210, and GSE42127). Rows represent different algorithm combinations, ranked in descending order by the mean C-index across validation cohorts (right-side bar plot); columns represent individual cohorts and their mean C-index, with color scale from blue to red indicating increasing C-index values. The RSF + SuperPC combination achieved the highest mean validation C-index (0.648) and was selected as the optimal model. **(B)** Principal component analysis (PCA) plot showing the distribution of samples from the TCGA and six GEO cohorts along the first two principal components (Dim1 and Dim2) following batch effect correction. Different colors represent different cohorts, and ellipses denote the 95% confidence interval for each cohort. Samples from all cohorts were well intermixed without evident cohort-specific clustering, indicating effective batch correction. **(C–I)** Kaplan-Meier survival curves for patients stratified into high-risk (red) and lowrisk (green) groups based on risk scores derived from the RSF + SuperPC model, using the optimal cutoff value, in the TCGA training cohort (C, n = 516) and six GEO validation cohorts: GSE13213 (D, n = 117), GSE26939 (E, n = 114), GSE29016 (F, n = 39), GSE30219 (G, n = 85), GSE31210 (H, n = 226), and GSE42127 (I, n = 133). Log-rank P values, hazard ratios (HR), and 95% confidence intervals are indicated on each plot; risk tables below each curve show the number of patients at risk at each time point.

**FIGURE 3:**
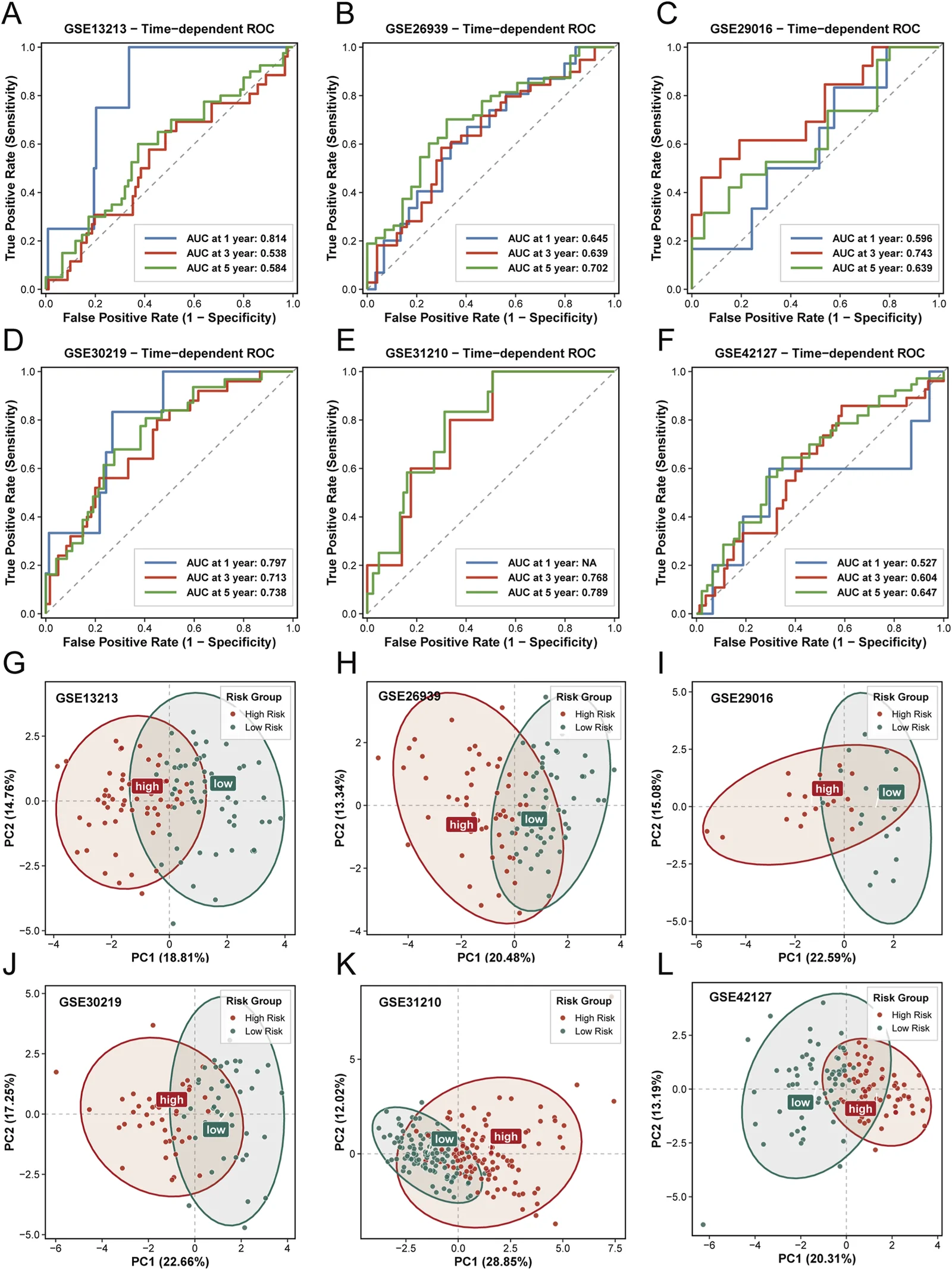
Predictive accuracy and patient stratification capacity of the core-gene risk model across six GEO validation cohorts. **(A–F)** Time-dependent ROC curves constructed using risk scores derived from the RSF + SuperPC model, for GSE13213 **(A)**, GSE26939 **(B)**, GSE29016 **(C)**, GSE30219 **(D)**, GSE31210 **(E)**, and GSE42127 **(F)**. Blue, red, and green curves represent ROC curves for 1-, 3-, and 5-year survival prediction, respectively, with corresponding AUC values indicated; the gray dashed line represents the reference line for random prediction (AUC = 0.5). **(G–L)** Sample distribution plots from principal component analysis (PCA) based on the expression of the 16 core genes, color-coded by risk group (high-risk: red; low-risk: green) as assigned by the risk model, for GSE13213 **(G)**, GSE26939 **(H)**, GSE29016 **(I)**, GSE30219 **(J)**, GSE31210 **(K)**, and GSE42127 **(L)**. Axes represent the first and second principal components (PC1, PC2) with their respective proportions of explained variance; ellipses denote the confidence interval for each risk group.

### Risk score associates with distinct immune microenvironments

We next examined whether the risk score captured variation in the tumour immune microenvironment. IPS differed between risk groups in three of the four CTLA4/PD1 categories, whereas the CTLA4-positive/PD1-negative category did not differ (Figures 4a–d). The direction of the IPS difference was not uniform across checkpoint categories, indicating that the risk score was associated with immunogenicity but did not support a single, treatment-specific response pattern. The continuous risk score correlated negatively with StromalScore (r = −0.17, q = 1.0 × 10^-4), ImmuneScore (r = −0.14, q = 0.0015) and ESTIMATEScore (r = −0.13, q = 0.0025), and positively with inferred tumour purity (r = 0.14, q = 0.0011; Figures 4e–h). Consistent with these modest but reproducible correlations, immune-deconvolution profiles from seven algorithms showed broad differences between the risk groups across lymphoid and myeloid cell populations (Figure 4i). High-risk tumours therefore exhibited a comparatively immune-depleted transcriptomic context, although the strength and direction of individual cell-type estimates varied by algorithm.

**FIGURE 4:**
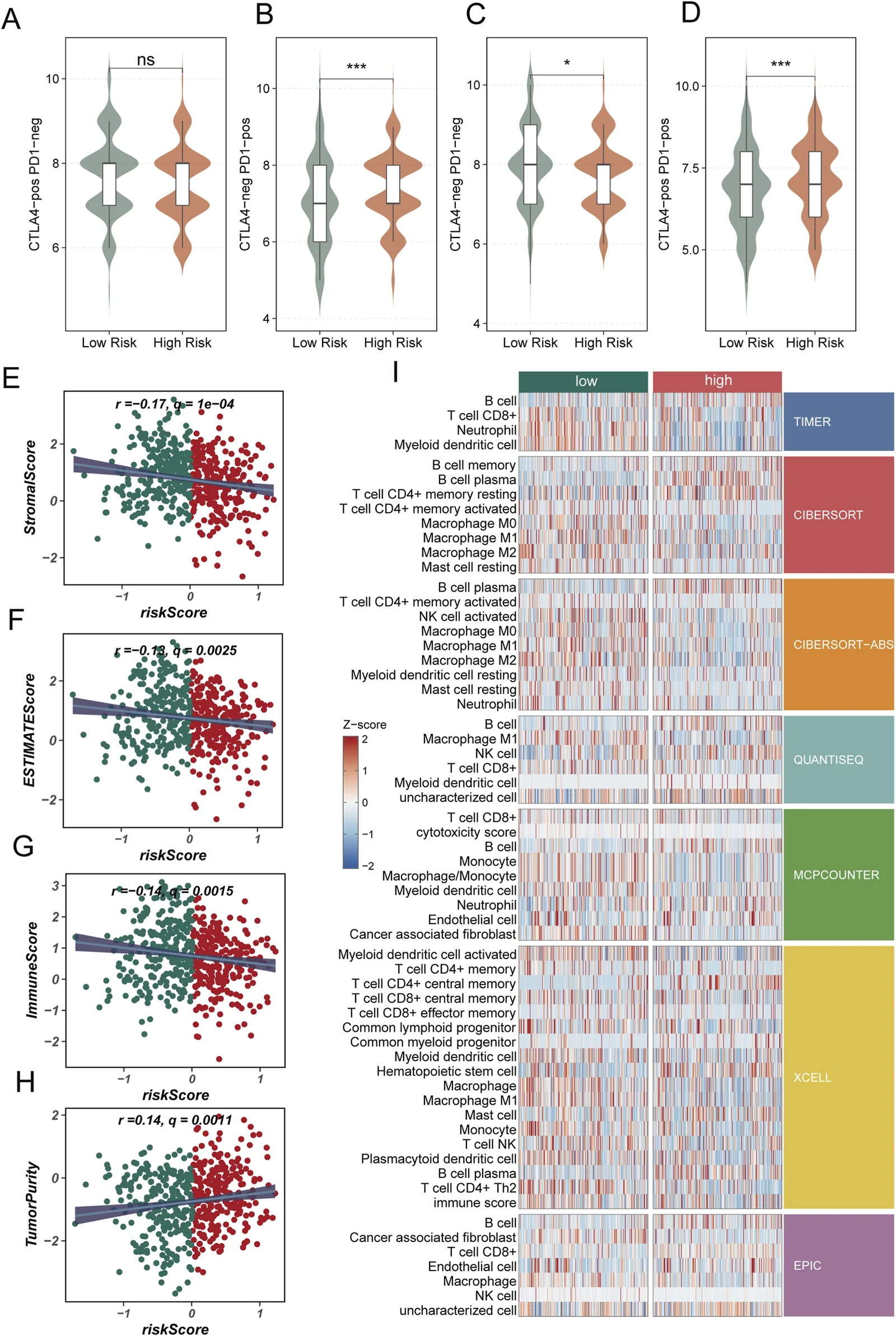
Association between the risk model and the tumor immune microenvironment and immunotherapy response. **(A–D)** Comparison of Immunophenoscores (IPS) between high- and low-risk groups in the TCGA-LUAD cohort across four immune checkpoint blockade scenarios: **(A)** CTLA4-negative/PD1-negative; **(B)** CTLA4-negative/PD1-positive; **(C)** CTLA4-positive/PD1-negative; **(D)** CTLA4-positive/PD1-positive. Box plots depict score distributions; group comparisons were performed using the Wilcoxon rank-sum test. **(E–H)** Spearman correlation analysis between risk score and ESTIMATE algorithm-derived tumor microenvironment scores: **(E)** stromal score; **(F)** immune score; **(G)** ESTIMATE composite score; **(H)** tumor purity. Each dot represents an individual patient; blue lines denote the fitted regression line with shaded 95% confidence intervals. **(I)** Comparison of immune cell infiltration levels between high- and low-risk groups in the TCGA-LUAD cohort, based on seven algorithms from the TIMER2.0 database (TIMER, CIBERSORT, CIBERSORT-ABS, QUANTISEQ, MCPCOUNTER, XCELL, EPIC), illustrating markedly enhanced immune infiltration in low-risk tumors versus an immune-desert phenotype in high-risk tumors.

### ATP2A2 emerges as the model gene most closely associated with risk

To identify a candidate gene for biological follow-up, we correlated each model gene with the RSF + SuperPC risk score. ATP2A2 showed the strongest positive association (r = 0.72, P < 0.001; Supplementary Figure S3), followed by PLAUR and TNFRSF12 A (both r = 0.70, P < 0.001). F10 was the only gene negatively correlated with risk (r = −0.26, P < 0.001). These results prioritized ATP2A2 for subsequent clinical and functional analyses. ATP2A2 was upregulated in LUAD relative to normal or adjacent lung in TCGA-LUAD and four of the five independent GEO datasets: GSE68465, GSE19188, GSE31210 and GSE63459 (Figures 5a,b,d–f). GSE11969 showed the opposite direction, with lower ATP2A2 expression in tumour samples (Figure 5c), indicating that the tumour-normal association was replicated in five of six cohorts but was not universal. Immunohistochemistry was consistent with higher ATP2A2 protein staining in tumour than in adjacent lung (Figures 5g,h; Supplementary Figure 8b). ATP2A2 expression also tracked with adverse clinicopathological features. Expression increased across histological grades in GSE68465 (Kruskal–Wallis P < 0.001), was higher in stage II than stage I tumours in GSE31210 (P < 0.001), and differed across stages I-IV in TCGA-LUAD (Kruskal–Wallis P = 0.0124; Figures 5i–k). Primary-tumour category showed the same pattern: ATP2A2 was higher in T2-T3 than T1 tumours in GSE30219 (P = 0.00211) and differed across T1-T4 tumours in TCGA-LUAD (P = 0.00232; Supplementary Figure S4). Representative IHC images from stages I-III further suggested increasing ATP2A2 staining with stage (Figure 5l, Supplementary Figure 8c). Together, these observations associate ATP2A2 with more advanced and less differentiated LUAD.

**FIGURE 5:**
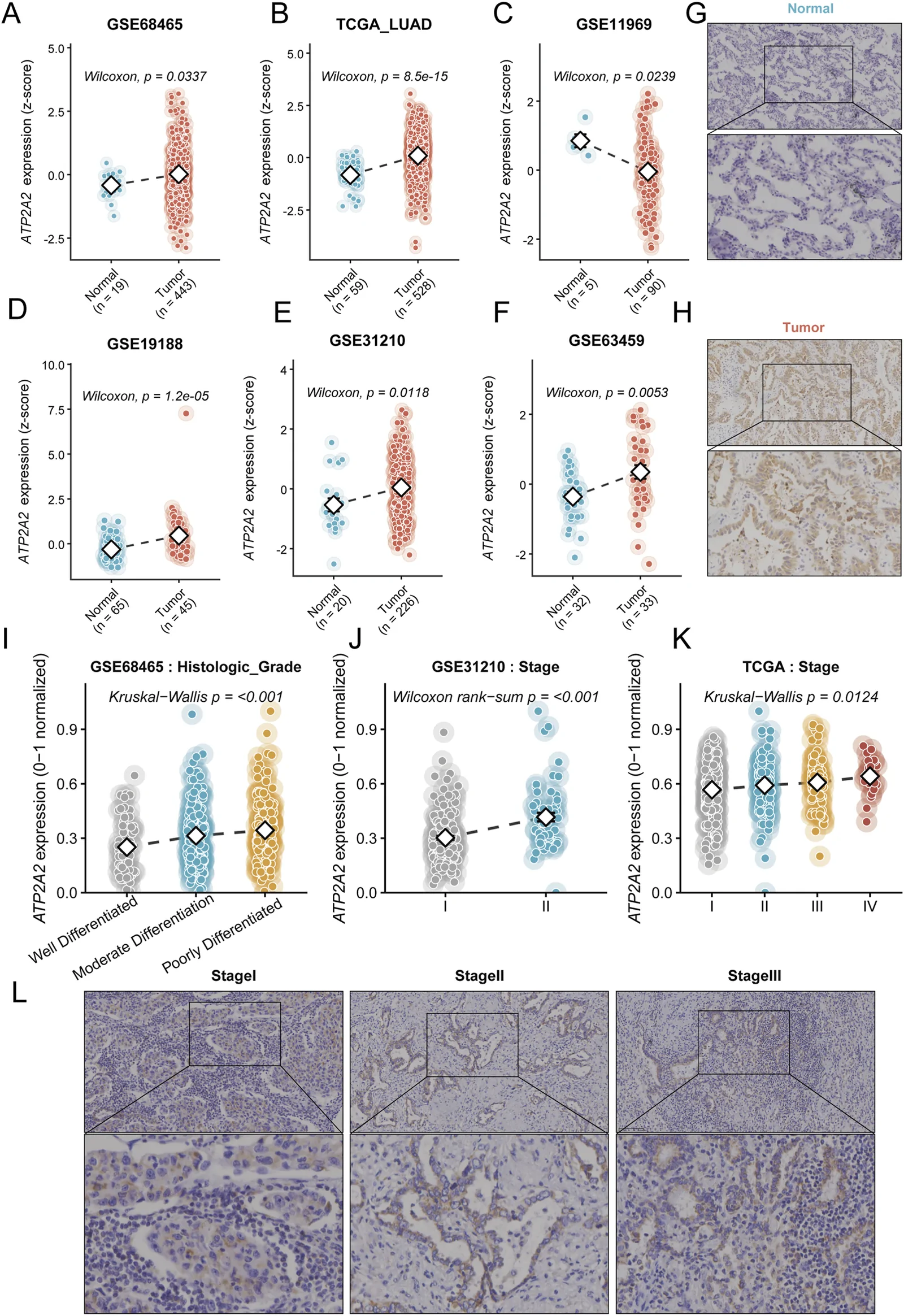
ATP2A2 is upregulated in LUAD and associated with tumor differentiation and clinical stage. **(A–F)** Expression of ATP2A2 in tumor versus normal/adjacent tissues in the TCGA-LUAD cohort and five independent GEO cohorts (GSE68465, GSE11969, GSE19188, GSE31210, GSE63459). **(G,H)** Representative immunohistochemical staining of ATP2A2 in LUAD tumor tissue and matched adjacent non-tumor tissue, showing higher protein expression in tumor tissue. **(I,J)** ATP2A2 expression stratified by histological differentiation grade. **(K)** ATP2A2 expression stratified by clinical stage. **(L)** Representative IHC images and quantification of ATP2A2 staining intensity in LUAD specimens across clinical stages I–III, showing a progressive increase in staining intensity with advancing stage. Data are presented as box plots or scatter plots with statistical comparisons as indicated (Wilcoxon rank-sum test or Kruskal–Wallis test).

### ATP2A2 promotes tumour progression and cell motility

High ATP2A2 expression was associated with shorter overall survival in two independent LUAD cohorts. In GSE26939, the high-expression group had an HR of 1.67 (95% CI 1.03–2.73; Cox P = 0.039; log-rank P = 0.037), and in GSE31210 the corresponding HR was 2.04 (95% CI 1.02–4.11; Cox P = 0.045; log-rank P = 0.040; Figures 6a,b). These analyses support ATP2A2 as a prognostic marker but do not establish independence from other clinical variables. We then evaluated whether ATP2A2 altered tumour-cell behaviour. Representative Transwell fields contained more crystal-violet-positive migrating and invading A549 cells after ATP2A2 overexpression than after empty-vector transfection (Figure 6c; Supplementary Figure 8a). This pattern was consistent with increased cellular motility following ATP2A2 overexpression and provided a functional link to the clinical associations with stage and T category.

**FIGURE 6:**
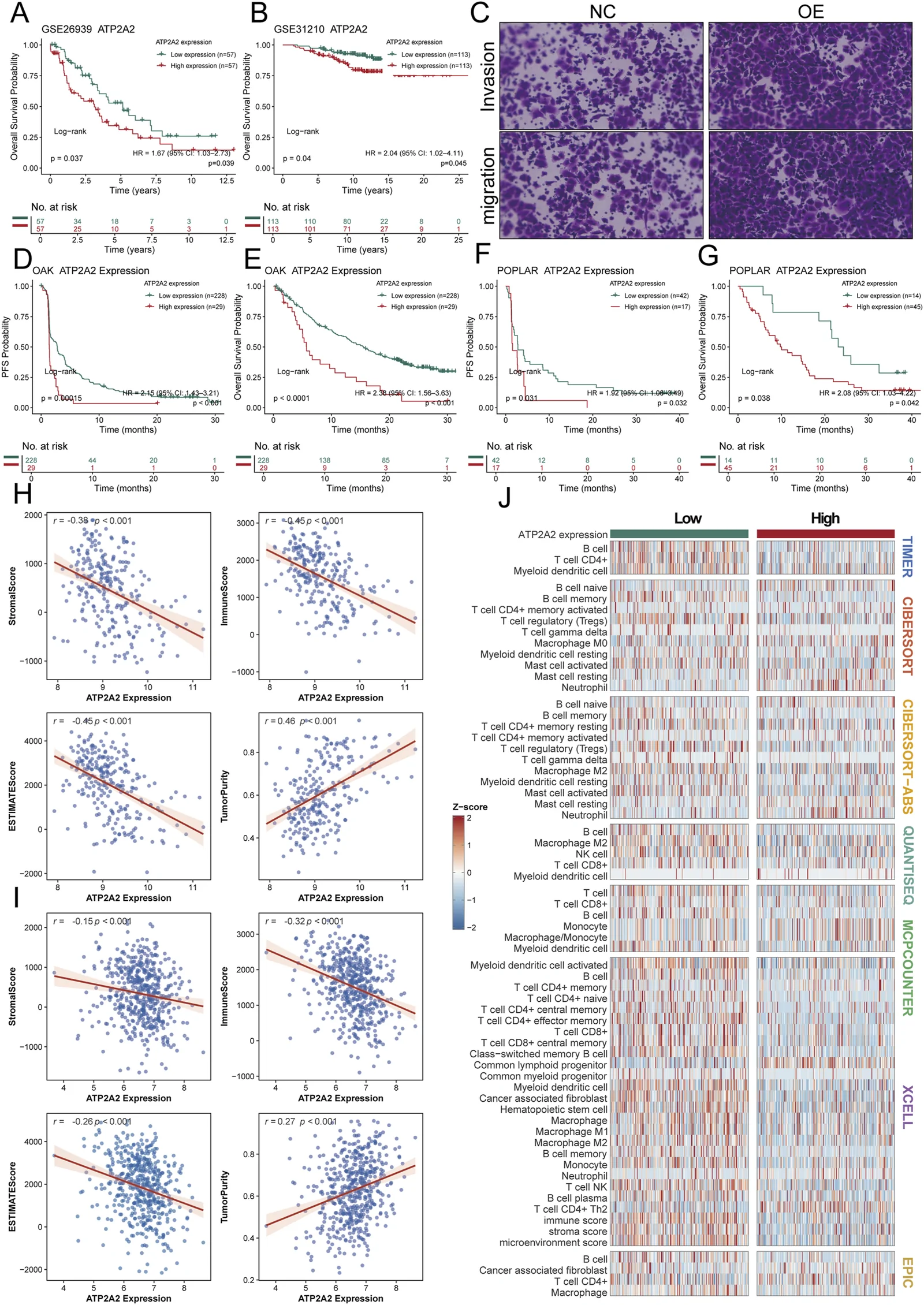
High ATP2A2 expression promotes LUAD invasion and migration and is associated with poor prognosis and an immunosuppressive tumor microenvironment. **(A,B)** Kaplan-Meier survival curves showing that patients with high ATP2A2 expression (red) had significantly shorter overall survival than those with low expression (green) in the GSE26939 **(A)** and GSE31210 **(B)** cohorts; between-group differences were assessed by the log-rank test, with the number of patients at risk at each time point shown below each curve. **(C)** Transwell migration and invasion assay results comparing A549 cells overexpressing ATP2A2 (OE) with negative control (NC) cells; representative fields of crystal violet–stained cells are shown (scale bar as indicated). **(D,E)** Kaplan-Meier curves for progression-free survival **(D)** and overall survival **(E)** in ATP2A2 high- versus low-expression groups in the OAK cohort; log-rank test was used to assess between-group differences, with the number of patients at risk shown below each curve. **(F,G)** Kaplan-Meier curves for progression-free survival **(F)** and overall survival **(G)** in ATP2A2 high- versus low-expression groups in the POPLAR cohort; log-rank test was used to assess between-group differences, with the number of patients at risk shown below each curve. **(H,I)** Scatter plots showing the correlation between ATP2A2 expression and stromal score (StromalScore), immune score (ImmuneScore), composite ESTIMATE score (ESTIMATEScore), and tumor purity (TumorPurity) in the OAK cohort **(H)** and the TCGA-LUAD cohort **(I)**; red solid lines represent linear regression fits, shaded areas represent 95% confidence intervals, and Pearson correlation coefficients (r) and p-values are annotated in each panel. **(J)** Heatmap showing differences in the infiltration levels of multiple immune cell subsets between ATP2A2 high- and low-expression groups in the TCGA-LUAD cohort, based on seven immune infiltration algorithms integrated in the TIMER database (TIMER, CIBERSORT, CIBERSORT-ABS, QUANTISEQ, MCPCOUNTER, XCELL, EPIC). Colors represent Z-score–normalized infiltration values, with red indicating higher infiltration and blue indicating lower infiltration; algorithm names corresponding to each panel section are labeled on the right.

### ATP2A2 associates with immune depletion and immunotherapy resistance

In the OAK immunotherapy cohort, high ATP2A2 expression was associated with shorter progression-free survival (HR = 2.15, 95% CI 1.43–3.21; log-rank P = 0.00015) and overall survival (HR = 2.38, 95% CI 1.56–3.63; log-rank P < 0.0001; Figures 6d,e). In POPLAR, overall survival was also shorter in the high-expression group (HR = 2.08, 95% CI 1.03–4.22; log-rank P = 0.038), whereas progression-free survival did not differ significantly by the log-rank test (P = 0.31; Figures 6f,g). The association therefore replicated for overall survival but was not consistent across all immunotherapy endpoints. ATP2A2 expression was inversely correlated with stromal, immune and composite ESTIMATE scores in both OAK (r = −0.38, −0.45 and −0.45, respectively; all P < 0.001) and TCGA-LUAD (r = −0.15, −0.32 and −0.26; all P < 0.001). Inferred tumour purity showed the opposite association (OAK, r = 0.46; TCGA-LUAD, r = 0.27; both P < 0.001; Figures 6h,i). Seven deconvolution algorithms further showed lower abundance estimates for multiple immune populations, including B-cell, T-cell and dendritic-cell subsets, in ATP2A2-high tumours (Figure 6j). These concordant associations linked high ATP2A2 expression to an immune-poor tumour context in both untreated and immunotherapy datasets.

### ATP2A2 impairs antitumour immune responses

IOBR-based analyses extended the ATP2A2-associated immune pattern across four deconvolution frameworks. In both OAK and TCGA-LUAD, increasing ATP2A2 expression coincided with lower ESTIMATE, MCPcounter, EPIC and QUANTISEQ estimates for several antitumour populations, including CD8 T cells, B cells and dendritic cells, and with higher inferred tumour purity (Supplementary Figures 5a, b). The same ordering revealed broad changes in immunomodulatory genes. ATP2A2 correlated negatively with several immune-recruiting chemokines and receptors, including CXCL13, CCL5 and CCL19, and with multiple immunostimulatory molecules, whereas a smaller set of inflammatory mediators, including CXCL1 and CXCL8, correlated positively (Supplementary Figures 5c, d). Checkpoint-associated immunogenicity scores were lower in the ATP2A2-high group across all four CTLA4/PD1 categories (Supplementary Figure 6a). Cancer-immunity-cycle analysis yielded a convergent pattern: ATP2A2 was negatively associated with antigen presentation, T-cell priming and activation, recruitment of CD8 T cells, NK cells and dendritic cells, recognition of malignant cells and tumour-cell killing (Supplementary Figure 6b). Several associations replicated in both OAK and TCGA-LUAD, including antigen presentation (r = −0.34 and −0.25), T-cell priming and activation (r = −0.34 and −0.22), dendritic-cell recruitment (r = −0.33 and −0.22), and T-cell recruitment (r = −0.33 and −0.24; adjusted P < 0.001 for each). These data place ATP2A2 within a coordinated immune-depleted transcriptional state rather than implicating a single immune-cell lineage.

### High ATP2A2 defines an epithelial-rich, CD8-poor microenvironment

We next examined ATP2A2-associated microenvironmental differences using the HRA001130 single-cell RNA-sequencing dataset. Unsupervised clustering and marker-based annotation identified 13 cell populations (Figures 7a,b; Supplementary Figure S7). These populations included lymphoid, myeloid, stromal and epithelial cell types. ATP2A2 expression was highest in ciliated and alveolar type II cells and was comparatively low in immune populations (Figure 7c). Samples were stratified according to the proportion of cells expressing ATP2A2. The ATP2A2-high group showed a descriptively lower proportion of T cells and a higher proportion of alveolar epithelial cells than the ATP2A2-low group (Figures 7d–f). Representative multiplex immunofluorescence images further showed fewer CD8-positive cells in ATP2A2-high, pan-cytokeratin-positive regions than in ATP2A2-low regions (Figure 7g). These observations were concordant with the bulk-tissue deconvolution results and associated high ATP2A2 with an epithelial-rich, CD8-poor microenvironment.

**FIGURE 7:**
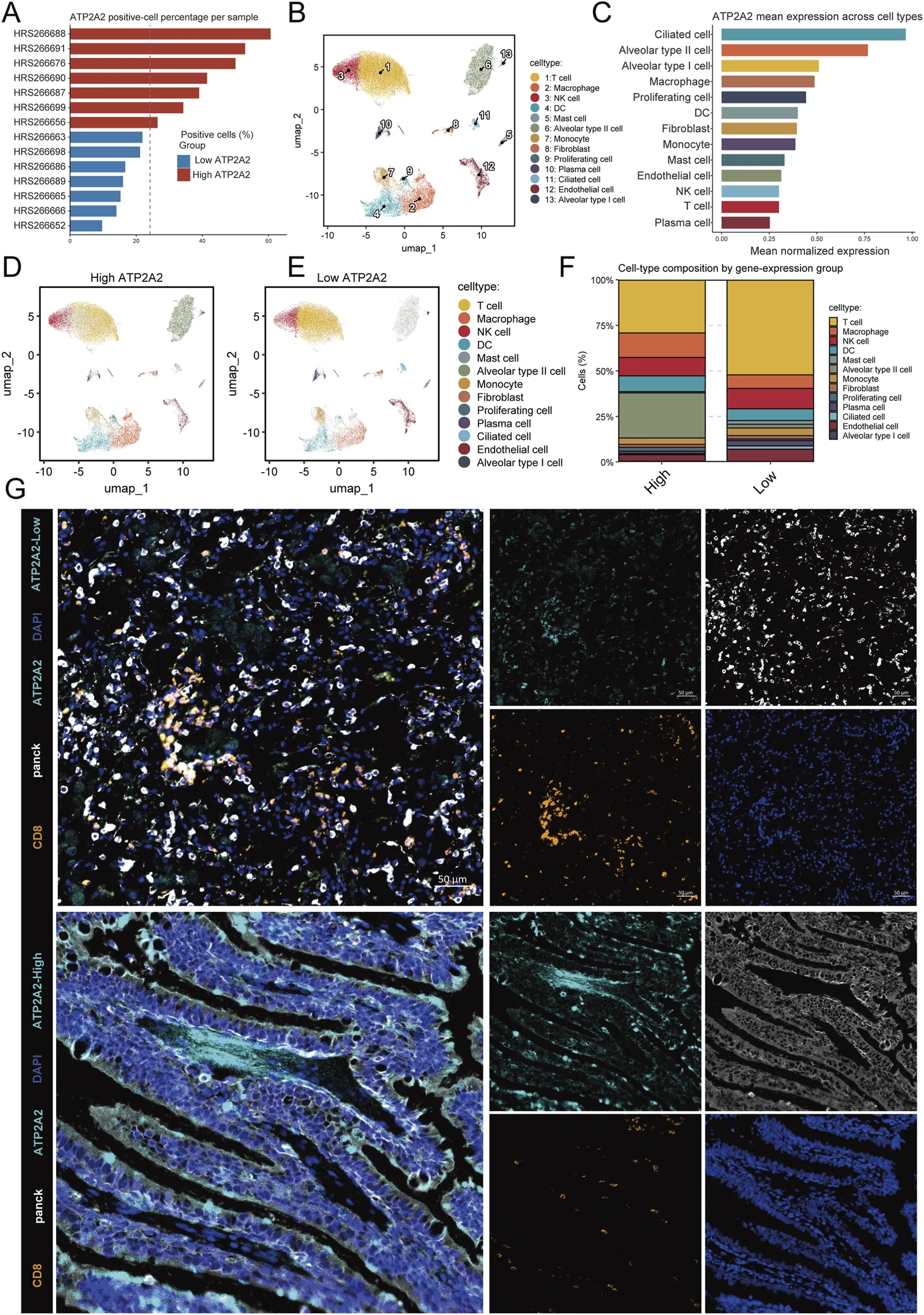
Single-cell profiling reveals that ATP2A2 expression reshapes the lung adenocarcinoma tumor microenvironment. **(A)** Percentage of ATP2A2-positive cells in each sample from the HRA001130 single-cell RNA-sequencing dataset. Samples were classified as ATP2A2-high or ATP2A2-low according to their sample-level ATP2A2-positive-cell fraction. Red denotes the ATP2A2-high group, and blue denotes the ATP2A2-low group. **(B)** UMAP representation of all cells, colored by marker-based cell-type annotation. Thirteen populations were identified: T cells, macrophages, natural killer cells, dendritic cells, mast cells, alveolar type II cells, monocytes, fibroblasts, proliferating cells, plasma cells, ciliated cells, endothelial cells and alveolar type I cells. **(C)** Mean normalized ATP2A2 expression across the 13 annotated cell populations (left) and the corresponding number of cells in each population (right). **(D,E)** UMAP representations showing the cell-type distributions within samples assigned to the ATP2A2-high group **(D)** and ATP2A2-low group **(E)**. **(F)** Relative cell-type composition of the ATP2A2-high and ATP2A2-low groups. The stacked bars show the proportion of each annotated population within each group. **(G)** Representative multiplex immunofluorescence images of human LUAD tissues stained for DAPI (blue), ATP2A2 (cyan), pan-cytokeratin (panCK; white) and CD8 (orange). The upper row shows an ATP2A2-low region, and the lower row shows an ATP2A2-high region. Individual fluorescence channels are shown on the right. Scale bar, 50 μm.

## Discussion

This study integrated 101 combinations of machine learning algorithms to construct and cross-validate a prognostic model for lung adenocarcinoma (LUAD) based on systemic inflammation–metabolic dysregulation–related genes, identifying ATP2A2 as a core hub gene. Chronic inflammation has long been regarded as a key driver of tumor evolution, sustained by continuous dynamic crosstalk among tumor cells, immune cells, and the inflammatory microenvironment (Elinav et al., 2013). A similar multi-algorithm integration strategy has previously been applied to construct prognostic models across multiple cancer types, screening robust prognostic signatures from 101 machine learning combinations and validating their predictive value in immunotherapy cohorts (Huan et al., 2024; Wang et al., 2026; Zhang et al., 2026), providing methodological precedent for the strategy adopted here. However, machine learning–derived multi-gene signatures are commonly criticized for their “black-box” nature—high predictive accuracy paired with weak mechanistic interpretability (Nayarisseri et al., 2021). This study addressed this limitation by anchoring the predictive power of ATP2A2 to concrete biological mechanisms through multi-layered validation using ESTIMATE, TIMER, single-cell transcriptomics, and multiplex immunofluorescence. Recent LUAD prognostic models have relied on limited gene sets or single validation platforms. In contrast, this study advances the field through three key innovations: a mechanistically-grounded gene panel selected from inflammation-metabolism dysregulation rather than statistical associations alone; systematic integration of 101 machine learning algorithms to identify the most robust model; comprehensive functional validation spanning bulk transcriptomics, immune deconvolution, single-cell resolution, and spatial protein analysis. This multi-omics integration directly addresses the interpretability gap in previous LUAD biomarker studies.

ATP2A2 encodes SERCA2, a core component of the endoplasmic reticulum (ER) calcium pump. The present results showing that its expression level is closely associated with remodeling of the tumor immune microenvironment can be understood through the lens of calcium homeostasis governing tumor cell fate. Disruption of ER calcium homeostasis is a key upstream event triggering autophagy and stress-related cell death pathways (Kania et al., 2015), and aberrant SERCA2 function has been shown to participate in regulating malignant progression in colorectal cancer (Fan et al., 2014). Pharmacological inhibition of SERCA can induce tumor cell death by depleting ER calcium stores (Sehgal et al., 2017), and this calcium-depletion process is closely linked to translocation of calreticulin to the cell surface—a molecular switch that determines whether tumor cell death is immunogenic (Obeid et al., 2007). Although SERCA inhibition and ER calcium depletion can promote calreticulin exposure during immunogenic cell death, this mechanism does not directly explain our finding that ATP2A2-high tumors were immune depleted. Further functional studies are required to determine whether elevated ATP2A2 alters ER calcium homeostasis and thereby suppresses antitumor immunity. Concurrently, suppression of T cell function by the tumor metabolic milieu (Chang et al., 2015) and mitochondrial dysfunction–driven T cell exhaustion (Scharping et al., 2021) offer complementary mechanistic explanations for the inverse correlation between ATP2A2 expression and immune cell infiltration observed via ESTIMATE and TIMER.

Single-cell data from HRA001130 further validated these findings at cell-subpopulation resolution. The immune subpopulation remodeling observed here is directionally consistent with previous single-cell atlases of non-small cell lung cancer, which identified profound tumor-driven remodeling of stromal cell phenotypes (Lambrechts et al., 2018). Cross-scale, cross-platform validation was completed at the protein level via multiplex immunofluorescence, demonstrating that ATP2A2-high tumors exhibit an epithelial-rich, CD8-poor microenvironment at both transcriptomic and spatial protein levels.

Regarding immunotherapy response prediction, this study calculated tumor immunogenicity scores (IPS) using the TCIA database and validated the predictive value of ATP2A2 expression for immunotherapy response in two independent clinical cohorts, OAK and POPLAR. IPS, as a composite metric quantifying tumor neoantigen burden and immunogenicity, has been shown to effectively distinguish potential responders to immune checkpoint inhibitors (Charoentong et al., 2017). OAK and POPLAR, as pivotal clinical trial cohorts evaluating atezolizumab efficacy, have been widely used as external validation datasets for biomarker studies (Rittmeyer et al., 2017; Fehrenbacher et al., 2016).

This study has two main limitations. First, the causal chain linking ATP2A2, calcium homeostasis, and immune microenvironment remodeling is currently supported mainly by expression correlation analyses and mechanistic inference from the literature, lacking direct evidence from *in vivo* gain-of-function/loss-of-function experiments. Second, the public database cohorts underlying this study carry inherent retrospective design biases and sample heterogeneity that cannot be fully corrected through algorithmic optimization alone.

In summary, this study positions ATP2A2 as a potential molecular hub linking systemic inflammation, metabolic dysregulation, and tumor immune microenvironment remodeling. Through multi-omics and multi-cohort cross-validation, it provides a novel biomarker candidate and mechanistic hypothesis for prognostic assessment and immunotherapy response prediction in lung adenocarcinoma. Future functional experiments are needed to establish causality, and prospective cohorts are needed to validate its clinical translational value.

## Data Availability

The original contributions presented in the study are included in the article/Supplementary Material, further inquiries can be directed to the corresponding authors.

## Supplementary material

SUPPLEMENTARY FIGURE S1 Waterfall plots detailing individual mutation patterns of representative inflammation-metabolism-related genes across the TCGA-LUAD cohort. **(A–F)** Supplementary oncoprint/waterfall plots showing sample-level mutation details (mutation type, variant classification, and co-occurrence patterns) for representative genes among the 117-gene panel in TCGA-LUAD tumor samples, complementing the overall mutational landscape presented in Figure 1A.

SUPPLEMENTARY FIGURE S2 Sample size distribution across the seven cohorts included in the analysis. **(A)** Bar plot showing the sample size and proportion of total samples (n = 1,230) contributed by the TCGA cohort and each of the six GEO cohorts (GSE13213, GSE26939, GSE29016, GSE30219, GSE31210, and GSE42127) prior to batch effect correction. **(B)** Time-dependent ROC curves for 1-, 3-, and 5-year survival prediction based on risk scores in the TCGA training cohort, with corresponding AUC values indicated. **(C)** Sample distribution plot from principal component analysis based on the expression of the 16 core genes in the TCGA training cohort, color-coded by high-risk (red) and low-risk (green) groups, with ellipses denoting the confidence interval for each group.

SUPPLEMENTARY FIGURE S3 Correlation between model gene expression and risk score in the TCGA-LUAD cohort. Scatter plots showing the correlation between expression levels of the 16 core model genes (ADM, PFKP, MT2A, PLAUR, CDA, PSMA3, TNFRSF12A, UBE2L6, LGALS3, ATOX1, ATP2A2, HSPA5, CP, IL13RA1, CFB, F10) and the risk score in the TCGA-LUAD cohort. Each dot represents an individual patient, colored by risk group (red, high-risk; green, low-risk). Linear regression fit lines with 95% confidence intervals are shown; Pearson correlation coefficients (r) and p-values are indicated in each panel.

SUPPLEMENTARY FIGURE S4 ATP2A2 expression increases with advancing primary tumor (T) stage. **(A)** ATP2A2 expression (0–1 normalized) in the GSE30219 cohort, comparing T1 versus T2–T3 tumors (two-sided Wilcoxon rank-sum test). **(B)**ATP2A2 expression (0–1 normalized) in the TCGA-LUAD cohort across T1, T2, T3, and T4 tumor stages (Kruskal-Wallis test). Each dot represents an individual sample; white diamonds indicate group means. P-values are shown in each panel.

SUPPLEMENTARY FIGURE S5 Correlation of ATP2A2 expression with tumor immune infiltration and immunomodulatory gene expression. **(A–B)** Heatmaps of immune infiltration scores calculated using the ESTIMATE, MCPcounter, EPIC, and QUANTISEQ algorithms in the OAK cohort **(A)** and the TCGA-LUAD cohort **(B)**; samples are arranged in ascending order of ATP2A2 expression (black curve, top), with the color bar above indicating high (red) versus low (blue) ATP2A2 expression groups, and significance of the correlation between each immune score and ATP2A2 expression indicated on the right (Pearson correlation; *p < 0.05, **p < 0.01, ***p < 0.001). **(C–D)** Heatmaps of chemokine, chemokine receptor, immunoinhibitory molecule, immunostimulatory molecule, and MHC molecule (D only) expression in the OAK cohort **(C)** and the TCGA cohort **(D)**, with side color bars indicating the direction of correlation (Direction) between each gene’s expression and ATP2A2 expression, along with the corresponding significance level.

SUPPLEMENTARY FIGURE S6 Correlation of ATP2A2 expression with immunotherapy response prediction scores and cancer immunity cycle activity. **(A)** Violin plots of TCIA scores in the ATP2A2 high- versus low-expression groups in the TCGA cohort under four immune checkpoint status categories (CTLA4-positive/PD1-positive, CTLA4-positive/PD1-negative, CTLA4-negative/PD1-positive, and CTLA4-negative/PD1-negative); green represents the low-expression group and orange represents the high-expression group; Wilcoxon rank-sum test, ***p < 0.001. **(B)** Bubble heatmap of Pearson correlations between ATP2A2 expression and the activity scores of the seven steps of the cancer immunity cycle in the OAK cohort (left column) and the TCGA cohort (right column); numbers within each cell represent the correlation coefficient r (color scale from red to blue denotes positive to negative r values), and circle size represents statistical significance (−log10(padj)); the legend on the right indicates the category (Step 1–Step 7) to which each step belongs.

SUPPLEMENTARY FIGURE S7 Marker gene expression across single-cell-annotated cell types. Dot plot showing the expression pattern of canonical marker genes across the 13 annotated cell types. Dot color represents the average expression level (red, high; blue, low), and dot size represents the percentage of cells expressing each gene (Percent Expressed).
